# Twenty‐Five‐Year Follow‐Up of Individuals With Intact Periodontium and Differing Susceptibility to Experimentally Induced Gingival Inflammation

**DOI:** 10.1111/jcpe.70163

**Published:** 2026-07-19

**Authors:** Leonardo Trombelli, Jenny Guidorzi, Dimitris N. Tatakis, Roberto Farina

**Affiliations:** ^1^ Research Centre for the Study of Periodontal and Peri‐Implant Diseases University of Ferrara Ferrara Italy; ^2^ Operative Unit of Dentistry Azienda Unità Sanitaria Locale (AUSL) Ferrara Italy; ^3^ Periodontics Department, College of Dentistry Texas A&M University Dallas Texas USA

**Keywords:** disease susceptibility, gingivitis, periodontitis, risk assessment

## Abstract

**Aim:**

To evaluate the periodontal condition of individuals with different susceptibility to dental biofilm–induced gingivitis 25 years after their classification as high responders (HR) or low responders (LR) to plaque.

**Methods:**

Forty‐eight systemically and periodontally healthy young adults who participated in a 21‐day experimental gingivitis trial in 2000–2001 and exhibited either markedly pronounced (HR, *n* = 24) or milder (LR, *n* = 24) gingival inflammation despite similar supragingival plaque exposure were invited to a follow‐up visit, during which a periodontal diagnosis was established.

**Results:**

Nine LR and eight HR individuals accepted to participate. Among the LR participants, six (67%) were diagnosed as periodontally healthy or with gingivitis, whereas 100% of HR participants were diagnosed with periodontitis (*p* = 0.009). Compared to LR, HR individuals presented higher bleeding on probing score (39.5% ± 13.8% vs. 15.0% ± 10.1%; *p* = 0.003) as well as a higher proportion of visibly inflamed sites (*p* < 0.001). Intergroup differences could not be explained by other variables, including full‐mouth plaque score, oral hygiene habits or adherence to supportive care.

**Conclusions:**

This study, based on a very limited sample size and therefore hypothesis‐generating rather than confirmatory, suggests that susceptibility to biofilm‐induced gingivitis may help identify individuals at different risks of periodontitis.

## 1 Introduction

The interaction between dental biofilm and host response may initially result in a reversible inflammatory gingival lesion that does not extend to the periodontal attachment apparatus (Page and Schroeder 1976). When clinically detected as bleeding on probing (BoP) with a full‐mouth prevalence > 10%, this condition has been defined as dental biofilm‐induced gingivitis (BIG) (Chapple et al. 2018; Trombelli et al. 2018). BIG is among the most prevalent inflammatory diseases, affecting individuals across all ages and geographic regions (Trombelli 2025).

In adults, the clinical signs and symptoms of BIG can be effectively managed (Peric et al. 2022; Ying et al. 2025; Tastan Eroglu et al. 2025; Biesbrock et al. 2026) through a tailored combination of risk factor control (Tatakis and Trombelli 2004; Trombelli and Farina 2013; Trombelli et al. 2021), behavioural interventions to enhance motivation and adherence to self‐performed biofilm control (Zhan et al. 2024; O'Malley et al. 2026) and professional mechanical plaque removal (Farina et al. 2026). BIG management improves oral health–related quality of life and reduces the systemic inflammation associated with BIG (Peric et al. 2022). But, more importantly, treatment is justified by the well‐established causal link between chronic gingival inflammation and periodontal attachment loss at both site level (Schätzle et al. 2003, 2004) and patient level (Joss et al. 1994; Ramseier et al. 2017), supporting BIG control as a key, sustainable strategy for the primary prevention of periodontitis (Chapple et al. 2015; Pattamatta et al. 2025).

Using the experimental gingivitis (EG) model, as originally proposed (Loe et al. 1965; Theilade et al. 1966) or subsequently modified (Jamjoom and Zahid 2025), consistent inter‐individual variability in gingival inflammatory response—independent of quantitative and qualitative aspects of plaque—has been repeatedly demonstrated (Abbas et al. 1986; Loe et al. 1965; Theilade et al. 1966; Wiedemann et al. 1979; Trombelli, Farina, et al. 2004; Trombelli et al. 2008; Schincaglia et al. 2017; Bamashmous et al. 2021). During the last 60 years, precision preventive periodontal medicine has focused on quantifying individual susceptibility to BIG, since improved understanding of this susceptibility can enable targeted allocation of resources and personalised strategies for BIG treatment, but also the early identification of individuals at risk for periodontitis, based on the hypothesis that susceptibility to BIG and periodontitis partly share risk factors (Trombelli 2004; Trombelli 2025). Further EG evidence supports the concept that the inflammatory response to plaque is a measurable individual trait that is also linked to periodontitis susceptibility: within‐person consistency in the severity of gingival inflammation has been observed both across time points within the same EG trial (Trombelli, Scapoli, Calura, and Tatakis 2006; Bamashmous et al. 2021) and across different EG trials (Trombelli et al. 2008); individuals resistant to periodontitis exhibit only mild inflammation even after prolonged oral hygiene abstention (van der Velden et al. 1985; Winkel et al. 1987), whereas periodontitis patients with reduced but healthy periodontium develop earlier, more extensive and more severe inflammation (van der Velden et al. 1985; Trombelli, Scapoli, Tatakis, and Minenna 2006). Although cross‐sectional studies corroborated the association between BIG susceptibility and periodontitis (Dietrich et al. 2006), this overarching hypothesis derived from EG models has never been substantiated by longitudinal (either short‐ or long‐term) observations.

The present study is based on a 25‐year follow‐up of two subsets of systemically and periodontally healthy young adults who, in a previous large‐scale randomised 21‐day EG trial, showed either a significantly more pronounced or milder gingival inflammatory responses (‘high responders’ [HR] and ‘low responders’ [LR], respectively) despite similar exposures to supragingival plaque. We hypothesised that 25 years after classification according to BIG susceptibility, HR individuals would show a higher prevalence and greater severity of periodontitis compared to LR individuals.

## 2 Materials and Methods

### 2.1 Experimental Design and Ethical Aspects

The present long‐term follow‐up cohort study consisted of a single follow‐up visit of individuals recruited among participants in a previous EG trial. Additional approval, beyond that initially obtained for the parent trial, was granted by the local Ethics Committee (protocol number: 134/2025/Oss/AUSLFe; approval date: 25 May 2025). All participants provided written informed consent.

### 2.2 Setting and Population

The study was conducted at the Operative Unit of Dentistry, Azienda Unità Sanitaria Locale, Ferrara, Italy. Participants were recruited from systemically and periodontally healthy volunteers who were previously enrolled in a split‐mouth, localised EG trial in the years 2000–2001 (Trombelli, Tatakis, et al. 2004). Specifically, participants who had been previously identified as HR (*n* = 24) or LR (*n* = 24), based on the severity of their gingival inflammatory response to experimentally induced plaque accumulation, were invited to participate in a single follow‐up visit between July and December 2025. Individuals were tentatively recalled using the telephone or e‐mail contacts that had been provided at the time of their participation in the EG trial; for those with missing/inactive contacts, a web search for new contacts was conducted.

### 2.3 Procedures and Parameters

During the follow‐up visit, the study procedures were performed by two expert clinicians (R.F. and J.G.) blinded as to the group (HR/LR), according to the following chronological sequence:

- Collection of medical and dental history.
- Collection of dental radiographs (if available).
- Administration of a battery of questionnaires to investigate (i) highest level of education, (ii) adherence to mediterranean diet (Gnagnarella et al. 2018), (iii) intensity of physical activity in the last 7 days (Craig et al. 2003), (iv) adherence to supportive periodontal care (SPC) and (v) frequency of self‐performed plaque control and oral hygiene devices used.
- Assessment of body weight (in kilograms) and height (in metres) for the calculation of the body mass index (BMI) as weight/height^2^.
- Assessment of the number and type of teeth present.
- Assessment of dental caries and any other pathologic conditions of the dental and oral mucosal tissues.
- Assessment, at six sites (i.e., mesio‐buccal, buccal, disto‐buccal, disto‐lingual, lingual, mesio‐lingual) for each tooth including fully erupted third molars, of:
  ○ Gingival index (GI) as evaluated according to the Trombelli, Tatakis, et al. (2004) modification of the method of Loë and Silness (1963).
  ○ Plaque score (PS), as evaluated using a modified version of the Plaque Control Record (O'Leary et al. 1972), based on evaluations at six sites (rather than four, as in the original publication) per tooth following the application of a plaque disclosing agent (Mira‐2‐Ton solution; Henry Schein, Krugg S.r.l.).
  ○ Probing depth (PD), from the gingival margin to the bottom of the sulcus/pocket using a probing force of about 20 N.
  ○ Gingival recession (REC), measured from the cemento‐enamel junction to the gingival margin during PD assessment.
  ○ Bleeding on probing (BoP), as evaluated during PD assessment according to Lang et al. (1986).
- Assessment of furcation lesions (Hamp et al. 1975) with a 2N Nabers probe (Hu‐Friedy Italy s.r.l., Milano, Italy).
- Assessment of tooth mobility (Miller 1950).

Probing measurements (PD, REC and BoP) were performed with a UNC15 probe (Hu‐Friedy Italy). No calibration session was held prior to the study to assess the level of intra‐ and inter‐examiner agreement in the evaluation of clinical parameters. However, clinical measurements were carried out with both expert examiners simultaneously present during the evaluations and using magnification loops (×2.5); one examiner performed the measurements while the second supervised the assessments, allowing immediate resolution of any discrepancies.

For each site, clinical attachment level (CAL) was calculated as the algebraic sum of PD and REC values. For each participant, full‐mouth plaque score (FMPS) and BoP score were calculated as the percentage of sites with plaque and BoP, respectively, and the BoP score/FMPS ratio was also calculated.

For each participant, probing parameters were used to formulate a diagnosis of periodontal health, BIG or periodontitis (Chapple et al. 2018; Trombelli et al. 2018; Papapanou et al. 2018; Tonetti et al. 2018). Gingivitis cases were further classified according to their extension (Chapple et al. 2018; Trombelli et al. 2018), while all periodontitis cases were qualified according to the staging framework (Papapanou et al. 2018; Tonetti et al. 2018). Participants with a diagnosis of periodontitis were informed about the indication to obtain a full‐mouth set of periapical radiographs and were offered the possibility to undergo the examination within the same follow‐up visit.

### 2.4 Statistical Analysis

The patient was the statistical unit. Comparisons were performed using Fisher's exact test or the Chi square test for categorical variables. All comparisons for continuous variables were performed using non‐parametric tests (Mann–Whitney U test). The relative risk (RR) for developing periodontitis in HR individuals was calculated using LR individuals as the reference group, using the Haldane–Anscombe correction for zero‐cell frequencies (Haldane 1956; Anscombe 1956). The level of statistical significance was set at 5%.

## 3 Results

### 3.1 Study Population

**FIGURE 1:**
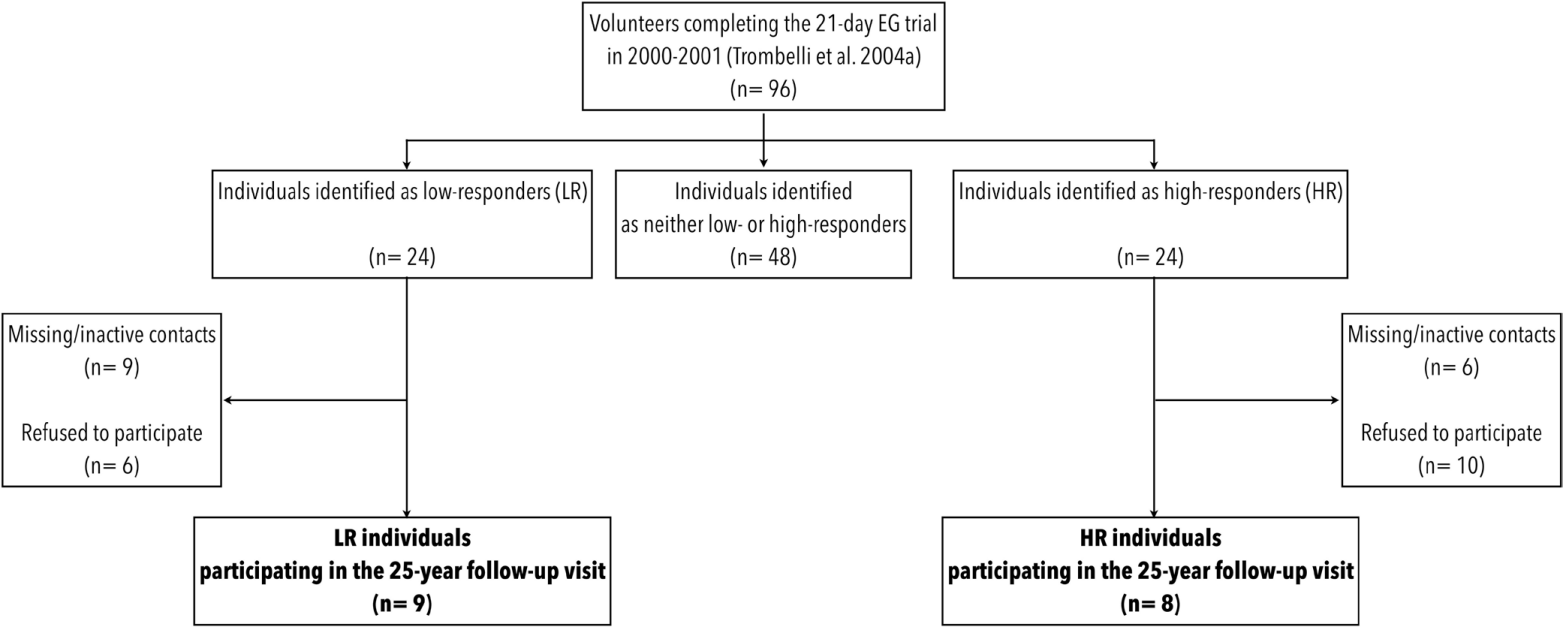
Flow of participants from the EG trial (Trombelli, Tatakis, et al. 2004) to the present 25‐year follow‐up study. EG, experimental gingivitis.

Flow of participants from the conclusion of the EG trial (Trombelli, Tatakis, et al. 2004) to the present follow‐up study is shown in Figure 1. Out of 48 individuals potentially eligible for this follow‐up study, 15 had missing or inactive contacts while 16 refused to participate, with the main reason being that they had moved away from the Ferrara metropolitan area. Among the participants who were successfully contacted, nine LR and eight HR individuals accepted to participate in the 25‐year follow‐up visit. None of them presented physical/mental disability or systemic/local conditions/diseases not compatible with a complete oral examination according to the study protocol. Also, none of them had recently undergone a dental/periodontal radiographic exam.

### 3.2 Assessment of HR and LR Participants at the 25‐Year Follow‐Up Visit

#### 3.2.1 General Health and Self‐Reported Exposures

Overall, the general health profiles of LR and HR groups remained consistent with the selection criteria applied 25 years earlier for inclusion in the EG trial (Trombelli, Tatakis, et al. 2004). Participants were in their mid‐adulthood, predominantly never‐smokers (except for one HR individual smoking less than 10 cigarettes/day) and reported no systemic diseases or medication use. Most individuals had a normal BMI. Educational attainment was high (degree or doctorate), approximately half adhered to a Mediterranean diet, and the majority reported engaging in physical activity within the previous 7 days. Both LR and HR groups retained most of their dentition, with a mean number of teeth exceeding 27 present in both groups. Only a few individuals reported tooth extraction, which in all cases was limited to a single episode and was not attributed to periodontitis or severe tooth mobility. No inter‐group differences were observed across the recorded characteristics (Table 1).

**TABLE 1:** General health status, lifestyles, self‐reported exposures and number of teeth as recorded in LR and HR individuals at the 25‐year follow‐up visit.

	LR (*n* = 9)	HR (*n* = 8)	*p*‐value (inter‐group difference)
Age^a^ (years)	48.7 (± 1.9; 46–51)	48.5 (± 1.5; 46–51)	0.704
Sex (male/female)	4/5	4/4	1
Smoking status (smokers/non‐smokers)	0/9	1/7	0.471
Self‐reported diabetes diagnosis (yes/no)	0/9	0/8	1
Body mass index (BMI) (< 18.5 underweight/18.5–24.9 normal weight/25.0–29.9 overweight/≥ 30 obese)	0/6/1/2	0/6/1/1	1
Highest education level (high school/graduate degree/doctorate)	0/7/2	0/5/3	0.620
Adherence to the mediterranean diet^b^ (low/high)	4/5	4/4	1
Intensity of physical activity in the last 7 days^c^ (inactive/active)	3/6	2/6	1
Teeth present^a^	27.7 (± 1.0; 26–29)	27.1 (± 1.5; 24–28)	0.472

Abbreviations: HR, high responders; LR, low responders (see text for details).

^a^ Values reported are mean (±SD; range).

^b^ Gnagnarella et al. (2018).

^c^ Craig et al. (2003).

#### 3.2.2 Oral Hygiene Habits, Performance, Local Factors and SPC Adherence

Oral hygiene habits and performance (as evaluated using FMPS) are reported in Table 2. FMPS values were high regardless of group (LR: 62.4% ± 13.1%; HR: 58.8% ± 17.2%). In both groups, no evidence of exposure to local risk factors for periodontitis, such as overhanging restorations or deep subgingival margins, was observed during oral screening. All participants were followed for SPC at private practice clinics, with the majority reporting professional dental cleaning once or twice annually. Most of them brushed their teeth 2–3 times daily and practiced no or only occasional interdental cleaning. No inter‐group difference in any of the listed parameters was observed (Table 2).

**TABLE 2:** Oral hygiene habits and performance as recorded in LR and HR individuals at the 25‐year follow‐up visit.

	LR (*n* = 9)	HR (*n* = 8)	*p* value (inter‐group difference)
Daily toothbrushing frequency (never or occasionally/1/2/≥ 3)	0/0/6/3	0/1/3/4	0.456
Daily interdental cleaning frequency (never or occasionally/1/2/≥ 3)	8/1/0/0	7/1/0/0	1
Yearly SPC session frequency (< 1/1–2/≥ 3)	1/8/0	2/6/0	0.576
FMPS^a^	62.4 (± 13.1; 45–86)	58.8 (± 17.2; 42–93)	0.412

Abbreviations: FMPS, full‐mouth plaque score (variation of plaque control record by O'Leary et al. 1972, based on six sites/tooth); HR, high responders; LR, low responders (see text for details); SPC, supportive periodontal care.

^a^ Values reported are mean (±SD; range).

#### 3.2.3 Periodontal Conditions

Periodontal parameters for LR and HR participants are presented in Table 3. Sixty‐seven percent of the LR group (6/9) was diagnosed with periodontal health or BIG, whereas 100% of the HR group developed periodontitis (*p* = 0.009). The relative risk of developing periodontitis in HR individuals was 2.7 times higher than that of LR individuals (RR = 2.70; 95% CI: 1.14–6.38; *p* = 0.024). Among those with periodontitis, no significant differences were found between the groups in the disease stage or extent, with most cases classified as localised stage II. The number of sites with varying severity of CAL loss in LR and HR groups is reported in Appendix S1. After being informed of the indication to obtain a full‐mouth series of periapical radiographs as a complementary diagnostic examination, all periodontitis cases, in both groups, decided to undergo the examination at their respective private practices, where they had been receiving routine dental care.

**TABLE 3:** Periodontal conditions of LR and HR individuals as recorded at the 25‐year follow‐up visit.

	LR(*n* = 9)	HR(*n* = 8)	*p* value(inter‐group difference)
Periodontal diagnosis^a^ (*n*)			
Health	2	0	0.009
Plaque‐induced gingivitis	4	0	
Periodontitis	3	8	
Periodontitis stage^a^ (*n*)			
I	1	2	0.279
II	1	6	
III	1	0	
IV	0	0	
Disease extent^a^ (*n*)			
Plaque‐induced gingivitis			
Localised	4	0	
Generalised	0	0	—
Periodontitis			
Localised	2	8	
Generalised	1	0	0.273
Bleeding on probing (BoP) score^b^, ^c^	15 (± 10.1; 2–36)	39.5 (± 13.8; 21–57)	0.003
Gingival Index (GI), within‐patient site distribution^d^, ^e^			
% sites with GI = 0	83.4 (± 11.4)	73.8 (± 23.9)	< 0.001
% sites with GI = 1	16.1 (± 10.9)	24.4 (± 23.9)	
% sites with GI = 2	0.3 (± 1.0)	1.8 (± 2.2)	
% sites with GI = 3	0.1 (± 0.4)	0 (± 0)	
Bleeding on probing (BoP)/full‐mouth plaque score (FMPS) ratio^e^	0.25 (± 0.19)	0.68 (± 0.24)	< 0.001

^a^ Chapple et al. (2018), Papapanou et al. (2018).

^b^ Lang et al. (1986) modification of the Index by Ainamo and Bay (1975).

^c^ Values reported are mean (±SD; range).

^d^ Trombelli, Tatakis, et al. (2004) modification of the Index by Loë and Silness (1963).

^e^ Values reported are mean (±SD).

BoP was significantly higher in the HR group (39.5% ± 13.8%; range: 21%–57%) than in the LR (15.0% ± 10.1%; range: 2%–36%) (*p* = 0.003). Additionally, the intra‐individual distribution of sites by GI score differed significantly between groups, with the HR group showing a lower proportion of GI = 0 sites and a higher proportion of GI = 1 or 2 sites (*p* < 0.001).

**FIGURE 2:**
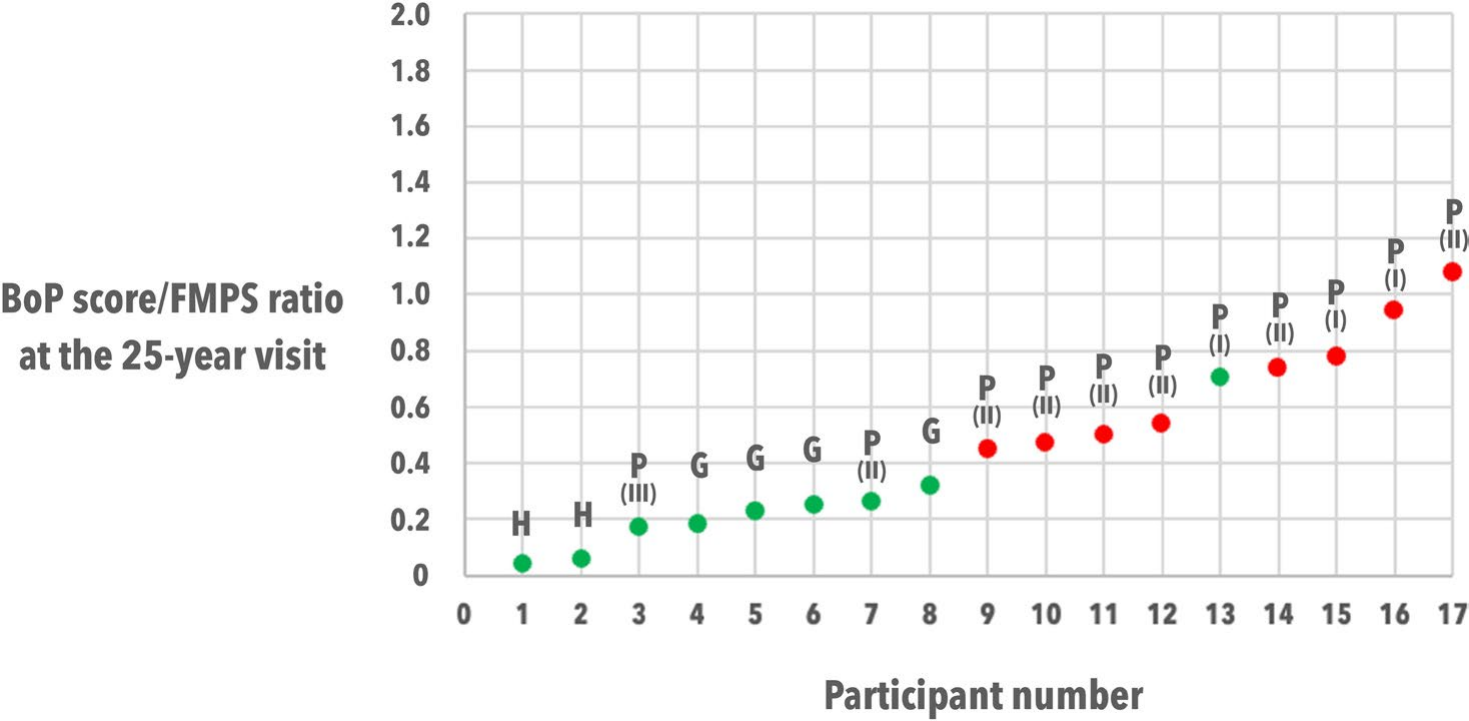
Distribution of LR and HR individuals (indicated by green and red dots, respectively) according to the BoP score/FMPS ratio at the 25‐year follow‐up visit. Labels above the dots indicate periodontal diagnosis (H: periodontal health; G: plaque‐induced gingivitis; P: periodontitis, with the stage reported in parentheses). BoP, bleeding on probing; FMPS, full‐mouth plaque score.

In the HR group, the BoP score/FMPS ratio was significantly higher than in the LR group (0.68 ± 0.24 vs. 0.25 ± 0.19, respectively; *p* < 0.001) (Table 3). Figure 2 shows individual BoP score/FMPS ratio and periodontal diagnosis in the HR and LR groups.

#### 3.2.4 Other Oral Diseases

No untreated interdental caries or other pathological conditions affecting hard or soft oral tissues were clinically detected at the follow‐up examination.

## 4 Discussion

The present study provides the first long‐term longitudinal data on periodontal outcomes in two subsets of systemically and periodontally healthy adults who, in a prior large‐scale 21‐day EG trial, showed either a pronounced (HR) or a mild (LR) gingival inflammatory response despite comparable supragingival plaque exposure. At the 25‐year follow‐up, individuals initially classified as HR showed a markedly higher prevalence of periodontitis (100%) compared with LR (33%). Furthermore, 25 years after the original EG trial that led to the characterisation of these individuals as HR and LR, the clinical parameters indicative of gingival inflammation, that is, BOP and GI, were significantly higher in the HR group compared to the LR group, despite similarly high plaque scores.

Individuals in the LR and HR groups showed a substantially lower and higher, respectively, prevalence rate and severity of periodontitis compared to estimates reported for mid‐adulthood (40–49 years) in an epidemiological study conducted in a metropolitan area in Italy (Aimetti et al. 2015). The significant differences in periodontitis prevalence between LR and HR were not paralleled by significant inter‐group differences in lifestyle, exposures to true or putative risk factors or oral hygiene habits and performance, all of which have been previously associated—albeit to varying degrees—with periodontitis risk (Shiau and Reynolds 2010; Boillot et al. 2011; Suvan et al. 2011; Eke et al. 2012; Keller et al. 2015; Lertpimonchai et al. 2017; Leite et al. 2018; Ferreira et al. 2019; Muniz et al. 2021; Stöhr et al. 2021; Wu et al. 2023; Fan et al. 2025; Shi et al. 2025; Farina and Hernandez‐Kapila 2026; Neapole et al. 2026).

Notably, after 25 years, the HR group exhibited significantly higher levels of periodontal (BoP score) and gingival inflammation (GI) compared to LR, in the absence of quantitative inter‐group differences in plaque (FMPS). This was even more pronounced when considering the BoP score/FMPS ratio (Table 3, Figure 2). Together with the markedly increased risk of developing periodontitis in HR versus LR individuals (RR = 2.70), this finding supports the concept of BIG susceptibility as an intrinsic individual trait influencing the future incidence of periodontitis, thereby reinforcing a central hypothesis underpinning a substantial body of periodontal research employing the EG model (Trombelli 2004). Although causal inference is precluded by the observational design, the hypothesis is biologically plausible given the well‐established role of chronic gingival inflammation in the initiation and progression of periodontitis (Lang et al. 1990; Joss et al. 1994; Suda et al. 2000).

Individual susceptibility to BIG (leading to classification as HR or LR) was derived from statistical modelling of data obtained in a 21‐day EG trial (Trombelli, Farina, et al. 2004). Briefly, gingival crevicular fluid volume at Day 21, considered the most objective indicator of inflammation, was standardised against cumulative plaque exposure (defined as the area under the curve of the plaque index over the 21‐day induction period) using linear regression. Residuals were then computed to capture the component of gingival crevicular fluid volume variability not explained by plaque accumulation. Trial participants were subsequently categorised as HR or LR based on the upper and lower quartiles of the standardised residual distribution. The use of standardised residuals from regression models is well established in research on complex diseases (Demissie et al. 2002; Tang et al. 2002; Ossenkoppele et al. 2020; Dumitrescu et al. 2020), as it allows isolation of unexplained outcome variability after accounting for known predictors. It is interesting to note that, although this statistical approach applied to the EG model allowed for the identification of two distinct BIG susceptibility patterns, the genetic, microbiological and immunological profiles, as well as the personality traits, social support and stress levels of HR and LR groups did not reveal differences that could explain their differing susceptibility to BIG (Trombelli, Tatakis, et al. 2004; Trombelli, Farina, et al. 2004; Trombelli et al. 2005; Trombelli et al. 2010; Scapoli et al. 2005; Scapoli et al. 2007).

Among those diagnosed with periodontitis, the entire HR group and two out of the three in the LR group were stage I‐II localised cases. In light of the data reported in Tables 1 and 2, these observations could be interpreted as suggesting that, even in the absence of systemic conditions associated with periodontitis, in the context of a healthy lifestyle, and with adherence to an SPC programme including at least 1–2 recall sessions per year, individuals with a higher susceptibility to BIG may have an increased likelihood of developing periodontitis, at least in mild forms, during mid‐adulthood. Conversely, individuals with lower susceptibility to BIG are less likely to develop periodontitis even in the presence of suboptimal self‐performed plaque control. These interpretations, however, should be considered with caution. No information could be retrieved on SPC providers and their training and expertise, the adopted SPC protocols and the level of patient adherence to their SPC programme. Moreover, both groups demonstrated remarkably high FMPS. Such plaque levels, which reflect findings from a single examination and do not necessarily represent the individuals' oral hygiene standards over the preceding 25 years, may appear inconsistent with the reported frequency of professional maintenance visits and oral hygiene practices; however, longitudinal evidence indicates that even under maintenance care in a general dental practice, patients often drift to poor plaque control over time (Axelsson and Lindhe 1981). The consistently high mean FMPS in both the LR and HR groups—suggestive of suboptimal oral hygiene compliance over the 25‐year period—precludes meaningful inference about how periodontal conditions, particularly in HR individuals, might have evolved under optimal plaque control. Notably, the only severe (stage III) periodontitis case occurred in the LR group. This finding suggests that low susceptibility to BIG alone did not predict long‐term periodontal outcomes in this individual and, to a lesser extent, in the other two LR participants with stage I–II periodontitis. In the absence of plausible explanations among the assessed periodontitis‐related exposures, further investigation of the systemic inflammatory profile and subgingival ecology of the LR group members who developed periodontitis, compared with the remaining group members, would be of interest.

The study has several limitations. First, the sample size was limited because of the difficulties in re‐contacting participants and securing their availability 25 years after the original EG trial. Although the observed difference in the prevalence of periodontitis between the groups is significant, the final small sample size limits the statistical power, reliability of the estimated effect size and generalisability of the findings. Therefore, the observed association between gingivitis susceptibility and periodontitis occurrence should be interpreted as hypothesis‐generating rather than confirmatory. Potential selection bias due to long‐term attrition cannot be excluded. However, the original EG population was characterised by a high degree of homogeneity in demographics and medical history (healthy, non‐smoking individuals aged 20–26 years) (Trombelli, Tatakis, et al. 2004). Furthermore, plaque accumulation and gingival inflammatory responses observed during the original 21‐day experimental gingivitis trial were comparable between the original HR and LR cohorts and the HR and LR subgroups participating in the present 25‐year follow‐up (Appendix S2). Although these observations cannot rule out attrition‐related bias, they suggest that the current HR and LR subgroups retained characteristics similar to those of the original cohorts. Although information on a series of potential confounders has been collected—some of them being only individually self‐reported—this may not be considered sufficiently detailed to capture the 25‐year exposures of the included subjects. Residual confounding from unknown/uncontrolled factors therefore remains possible, and the observed association between BIG susceptibility and periodontitis occurrence should be interpreted as an association rather than as evidence of a causal relationship. The limited sample size (*n* = 17) in relation to (i) the high number of determinants that should be entered into the model in addition to subject group (HR/LR) and (ii) the unfavourable distribution of individuals for some parameters prevented us from performing a multivariate analysis. Although experts and blinded and jointly involved in all assessments, the two examiners were not involved in a calibration exercise.

The findings of the present study suggest that susceptibility to BIG (as assessed according to the methodology described by Trombelli, Tatakis, et al. 2004) is associated with the occurrence of periodontitis over a 25‐year period and seems to indicate that lower BIG susceptibility may help identify individuals with a favourable likelihood of maintaining periodontal health or a gingivitis status, as well as distinguish those at increased risk of developing periodontitis. Given the very limited sample size, these findings should be regarded as hypothesis‐generating. Further investigations in larger cohorts are warranted to validate these observations and to explore the potential role of BIG susceptibility in contemporary preventive periodontal medicine.

## Author Contributions

L.T., D.N.T. and R.F. conceptualised the study and finalised the manuscript. R.F. and J.G. performed the clinical procedures. J.G. prepared the study dataset, and R.F. performed the statistical analysis.

## Conflicts of Interest

The authors declare no conflicts of interest.

## Supporting information


**Appendix S1:** Number of sites with varying severity of CAL loss in HR and LR individuals.

**Appendix S2:** Plaque accumulation (PlI) and severity of gingival inflammation (mGI, AngBS, GCF volume) at the conclusion of the 21‐day experimental gingivitis trial for test quadrants of all LR and HR individuals (data reported from Trombelli, Tatakis, et al. 2004) and LR and HR individuals participating in the 25‐year follow‐up visit. For this table, descriptive statistics (mean ± standard deviation or median and interquartile range) were kept consistent with those used in the study by Trombelli, Tatakis, et al. (2004).

## Data Availability

The data that support the findings of this study are available from the corresponding author upon reasonable request.
