# Supplementary material for: Twenty‐Five‐Year Follow‐Up of Individuals With Intact Periodontium and Differing Susceptibility to Experimentally Induced Gingival Inflammation
Source: J Clin Periodontol. 2026 Jul 19;53(9):1393–402. doi: 10.1111/jcpe.70163 (PMC13477845; doi:10.1111/jcpe.70163)
Supplement: Supplementary file 1 [file JCPE-53-1393-s001.docx]

**Appendix S2.** Plaque accumulation (PlI) and severity of gingival inflammation (mGI, AngBS, GCF volume) at the conclusion of the 21-day experimental gingivitis trial for test quadrants of all LR and HR individuals (data reported from Trombelli et al. 2004a) and LR and HR individuals participating in the 25-year follow-up visit. For this Table, descriptive statistics (mean ± standard deviation or median and interquartile range) were kept consistent with those used in the study by Trombelli et al. (2004a).

|  | **LR** | | **HR** | |
| --- | --- | --- | --- | --- |
|  | **all individuals**  **(n= 24;**  **Trombelli et al. 2004)** | **individuals participating in the 25-year follow-up**  **(n= 9)** | **all individuals**  **(n= 24; Trombelli et al. 2004)** | **individuals participating in the 25-year follow-up**  **(n= 8)** |
| **Plaque Index (PlI)^a^** | 1.65  (± 0.37) | 1.59  (± 0.35) | 1.73  (± 0.33) | 1.83  (± 0.28) |
| **Modified Gingival Index (mGI)^b^** | 0.50  (0.33 – 0.58) | 0.50  (0.33 – 0.50) | 0.83  (0.67 – 1.00) | 0.83  (0.46 – 1.00) |
| **Angulated Bleeding Score (AngBS)^c^** | 0.33  (0 – 0.75) | 0.33  (0.33 – 0.83) | 0.67  (0.42 – 1.0) | 0.58  (0.33 – 1.00) |
| **Gingival crevicular fluid (GCF) volume^d^** | 0.22  (± 0.07) | 0.15  (± 0.05) | 0.46  (± 0.13) | 0.35  (± 0.06) |

^a^ Furuichi et al. 1992 modification of the Index by Silness & Löe 1964

^b^Trombelli et al. 2004a modification of the Index by Löe & Silness 1963

^c^ Trombelli et al. 2004a modification of the Index by Van der Weijden et al. 1994

^d^ in microliters
