# Supplementary material for: Twenty‐Five‐Year Follow‐Up of Individuals With Intact Periodontium and Differing Susceptibility to Experimentally Induced Gingival Inflammation
Source: J Clin Periodontol. 2026 Jul 19;53(9):1393–402. doi: 10.1111/jcpe.70163 (PMC13477845; doi:10.1111/jcpe.70163)
Supplement: Supplementary file 2 [file JCPE-53-1393-s002.docx]

**Appendix S1.** Number of sites with varying severity of CAL loss in HR and LR individuals.

|  | **LR group** | | | | | | | | | **HR group** | | | | | | | |
| --- | --- | --- | --- | --- | --- | --- | --- | --- | --- | --- | --- | --- | --- | --- | --- | --- | --- |
| Severity of CAL loss | #1 | #2 | #3 | #4 | #5 | #6 | #7 | #8 | #9 | #1 | #2 | #3 | #4 | #5 | #6 | #7 | #8 |
| 1 mm  2 mm  3 mm  4 mm  ≥ 5 mm | 0  0  0  1  0 | 0  0  0  0  0 | 0  0  0  0  1 | 0  0  0  0  0 | 0  0  2  0  0 | 0  0  0  0  0 | 0  1  0  0  0 | 21  4  2  9  1 | 42  4  0  0  0 | 12  4  1  1  0 | 2  0  4  2  0 | 8  0  0  0  0 | 3  0  2  0  0 | 4  1  2  6  0 | 5  0  0  0  0 | 8  3  1  0  0 | 0  0  4  0  0 |

CAL: Clinical attachment level; HR: high responders; LR: low responders (see text for details)
